# Seeing Is Believing: Gap Junctions in Motion

**DOI:** 10.3390/biology10060494

**Published:** 2021-06-02

**Authors:** Xinbo Li

**Affiliations:** Casey Eye Institute, Oregon Health and Science University, Portland, OR 97239, USA; lixinb@ohsu.edu

Gap junctional intercellular communication (GJIC) channels between cells are composed of connexin proteins that form hexamers (connexons) in adjacent plasma membranes. Connexons (hemichannels) in apposing membranes dock to create pores allowing the passage of ions and small molecules with a molecular weight of less than 1000 Daltons, such as water, amino acids, nucleotides, ATP, calcium, cAMP, and IP3. To date, there are 20 mouse connexin genes and 21 human connexin genes; three pannexin genes, namely, pannexin1–3, have also been cloned and widely studied. In addition, the invertebrate gap junctions, called innexins, have also attracted many researchers’ attention and the studies involving innexins are expanding rapidly [1,2,3].

These gap junction proteins are differentially expressed in different organs, tissues, or cells and show additional functional channel selectivity with each other. GJIC plays a pivotal role in maintaining the physiological function of cells and tissues, which include cell differentiation, cell proliferation, apoptosis, cell development, and tissue homeostasis. GJIC can be measured using small molecular weight dyes such as Lucifer yellow or neurobiotin combined with single-cell microinjection, scrape loading or preloading methods, or by using the dual patchclamp technique to measure the channel conductance in paired cells. Individual cells often express more than one connexin type, and gap junctions are widely expressed in nearly all tissues. For example, different gap junction proteins are expressed in different cellular populations of the central nervous system (CNS). Connexin36 (Cx36) is widely expressed in neurons, whereas astrocytes express Cx26, Cx30, and Cx43, oligodendrocytes express Cx29, Cx32, and Cx47. Multiple connexins are widely expressed in the mammalian visual system; the expression of Cx30.2, Cx36, Cx45, and Cx57 have been previously described in the retinal neurons, while the lens is connected by gap junctions comprised of Cx46 and Cx50 [4,5].

It is well established that gap junctions are highly dynamic structures, and the states of gap junction coupling are heavily regulated. Modulation of the gap junctional coupling state is proposed to underlie rapid shifts in cellular network connectivity. Further, gap junctions exhibit rapid alteration in the coupling state in response to a stimulus, and gap junction proteins generally have a high turnover rate (for Cx36 and Cx43, a half-life around 1–3 hrs). Gap junctions can be regulated at the DNA, RNA, or protein level. Transcriptional control is one of the most important mechanisms for regulating connexin gene expression. Up to now, several transcription factors have been reported to regulate the related connexin gene expression, and interestingly, the Cx43 (GJA1) gene can use an alterative translation site to generate multiple N-terminal truncated c-terminal preserved forms of Cx43, such as GJA1-20k. Further, posttranslational modifications in gap junction proteins, such as phosphorylation, ubiquitination, and glycosylation play pivotal roles in regulating channel gating, turn over, cellular localization, trafficking, and protein–protein associations [6,7]. However, it appears that the posttranslational modification states in many connexin proteins, including Cx25, Cx29, Cx30, Cx31.9, Cx47, and Cx57, are not well established.

Gap junctions are emerging as multimolecular protein complexes and their regulation is mediated in part by their associated proteins. The first well-established Cx43 interacting protein is ZO-1 (zonula occludens-1, also called tight junction protein-1 or TJP1). The association of Cx43 with ZO-1 involves the c-terminal PDZ domain-binding motif of Cx43 (DLEI) and the second PDZ domain of ZO-1, and deletion of the last amino acid residue “I” (isoleucine) in Cx43 can eliminate its association with ZO-1. To date, nearly a dozen connexins have been shown to interact with the second PDZ domain of ZO-1 [8]. However, there is one sole exception, that is the case of Cx36, which represents the major component of electrical synapses in the central nervous system. Indeed, Cx36 and Cx35 (the fish ortholog of mammalian Cx36) contain a highly conserved “SAYV” PDZ domain-binding motif at their extreme c-terminus, via the SAYV PDZ domain-binding motif, while Cx36 and Cx35 all interacted with the first PDZ domain of ZO-1 [9]. Interestingly, the kinetics of the association of Cx35/Cx36, or Cx43 with ZO-1 was investigated by the surface plasmon resonance (SPR) using the separate PDZ domain of ZO-1 fusion proteins as well as Cx35/Cx36 or Cx43 C-terminal peptides. The SPR analyses showed that the kinetic of Cx36-PDZ1 of ZO-1 binding is much smaller than the Cx43-PDZ2 of ZO-1 binding, indicating the Cx36 (Cx35)-ZO-1 interaction is of lower affinity, which may represent the more dynamic feature of electrical synapses composed by Cx36 [10,11].

Changes in gap junction protein expressions are observed in some disease conditions. It has been shown that Cx43 is upregulated under inflammatory disease conditions, such as sepsis. Under inflammatory conditions, the increased expression of Cx43 may open the GJIC as well as connexin hemichannels and promote the release of more ATP and cell cytokine, resulting in a burst of cytokine storm and subsequently causing severe damage to the cells and tissues affected. Therefore, blockade or inhibition of GJIC or connexin hemichannels may offer beneficial effects in the treatment of inflammatory diseases [12]. Traditional gap junction channel inhibitors have shown therapeutic effects, however, it has also been argued that these gap junctional inhibitors may show some nonspecific effects. Therefore, the use of synthetic mimetic peptides that specifically block or inhibit GJIC or connexin hemichannels may provide a novel way in the treatment of inflammatory disease conditions.

Gap junction gene mutations are associated with a variety of human disease conditions such as hearing loss (Cx26, Cx30, Cx31); cataracts (Cx46, Cx50); X-linked Charcot-Marie-Tooth (CMTX) disease (Cx32), and oculodentodigital dysplasia (Cx43), importantly, the same gap junction genes with mutations at different sites may cause two completely different diseases, indicating the complexity nature of gap junctions [13,14,15]. Mutations in Cx26 account for about 50% of nonsyndromic hearing loss, which is one of the highest incidences of any given genetic disease [16]. Therefore, there is an urgent need to investigate whether the gene therapy strategy using a variety of recently developed techniques such as CRISPR/Cas9 gene editing may have a beneficial effect on the treatment of hearing loss in children carrying a connexin gene mutation. Considering that many connexin gene mutations in patients are single base-pair mutations, recently discovered base editing and prime editing may correct the single mutant base pair and offer a new solution or restore the hearing loss in children in the near future, especially due to its high efficiency and less off-target effects compared with CRISPR/Cas9 system [17].
